# Photonic Crystal Circular Nanobeam Cavity Laser with Type-II GaSb/GaAs Quantum Rings as Gain Material

**DOI:** 10.1038/s41598-020-61539-5

**Published:** 2020-03-16

**Authors:** Hsiang-Ting Lin, Kung-Shu Hsu, Chih-Chi Chang, Wei-Hsun Lin, Shih-Yen Lin, Shu-Wei Chang, Yia-Chung Chang, Min-Hsiung Shih

**Affiliations:** 1 Research Center for Applied Sciences (RCAS), Academia Sinica, Taipei, 11529 Taiwan; 2 Department of Photonics and Institute of Electro-optical Engineering, National Chiao Tung University, Hsinchu, 30010 Taiwan; 3 Graduate Institute of Electronics Engineering, National Taiwan University, Taipei, 10617 Taiwan; 4 Department of Photonics, National Sun Yat-sen University, Kaohsiung, 80424 Taiwan

**Keywords:** Semiconductor lasers, Nanocavities, Photonic crystals, Quantum dots

## Abstract

The optical emission from type-II semiconductor nanostructures is influenced by the long carrier lifetime and can exhibit remarkable thermal stability. In this study, utilizing a high quality photonic crystal circular nanobeam cavity with a high quality factor and a sub-micrometer mode volume, we demonstrated an ultra-compact semiconductor laser with type-II gallium antimonide/gallium arsenide quantum rings (GaSb/GaAs QRs) as the gain medium. The lasing mode localized around the defect region of the nanobeam had a small modal volume and significant coupling with the photons emitted by QRs. It leads the remarkable shortening of carrier lifetime observed from the time-resolved photoluminescence (TRPL) and a high Purcell factor. Furthermore, a high characteristic temperature of 114 K was observed from the device. The lasing performances indicated the type-II QRs laser is suitable for applications of photonic integrated circuit and bio-detection applications.

## Introduction

Semiconductor nanostructures based on gallium antimonide (GaSb) and gallium arsenide (GaAs) have aroused considerable research interest because of their type-II band alignments and distinct material properties from those of the known InAs/GaAs based systems^1,2^. The staggered type-II band alignment leads to spatially indirect transitions of carriers and several unique optical properties such as the long carrier lifetime, wide coverage of emission wavelengths in the infrared (IR) regime, and stable emission less sensitive to the thermal effect. The optical characteristics and carrier dynamics of GaSb/GaAs quantum dots (QDs) systems have been investigated^3–8^ and utilized in various applications including lasers^9–13^, optical memories^14–18^, bioimages^19^, and light emitting diodes^20,21^. However, the spatially indirect transition of carriers in type-II band structures also lowers the radiative recombination probability and hence limits the corresponding emission efficiency^22^. Recently, with the optimized source flux ratio during molecular beam epitaxy (MBE)^23–27^, the growth of GaSb/GaAs quantum rings (QRs) has been demonstrated, and these nanostructures exhibit the more intense photoluminescence (PL) than GaSb/GaAs QDs do^28,29^. The QRs have the less abrupt GaSb/GaAs interfaces but larger surface area than QDs do, which improve the wave function overlap between electrons and holes and therefore boost up the radiative recombination significantly^30^. The luminescence from coupled GaSb/GaAs QRs at room temperature can be even comparable to that of type-I InAs QDs^29^, indicating that GaSb QRs might play a role in the applications of laser diodes and light-emitting diodes^28,31^.

Recently the novel low-dimensional gain materials such as QW^32,33^, QD^12,13,34–38^, and two-dimensional materials^39,40^ were integrated with compact optical cavities for lasing devices under continuous-wave^32,35,36,40^ and electrical injection pumping^34,37,38^ conditions. In this study, we demonstrated a photonic crystal (PhC) circular nanobeam defect cavity^41^ laser with the type-II GaSb/GaAs QRs as gain medium, for first time. The small type-II QRs laser exhibit several unique properties such as longer carrier and photon lifetime, high characteristic temperature, compare to similar sized type-I QDs or QRs lasers. Leveraging the confinement of light due to photonic bandgap, the PhC-based lasers benefit from the low radiation loss (high radiation quality factor^42–48^), small optical modal volume^49–51^, high Purcell factor^52–54^, and low lasing threshold^55,56^ which are suitable for chip-scale photonic integrations^57–60^. The nearly diffraction-limited confinement of resonant modes in PhC lasers significantly enhance the light-matter interactions, but these devices also suffer from the issue of thermal stability due to the fabricated photonic structures which impede heat conduction. As a result, the performance of the lasing devices is sensitive to temperature, which limits their practical usages. It was reported that the gain medium based on type-II nanostructures can sustain thermally stable photon emissions^61–64^. The staggered type-II band structures efficiently suppress the nonradiative Auger recombination which quenches the light emission at high pumping intensity^12,61–64^. Therefore, integrating PhC nanocavities with type-II QRs may provide a platform for advanced lasing light source with high thermal stability.

For the realization of lasers based on type-II nanostructures, an epitaxial wafer of GaSb/GaAs QRs with a peak emission wavelength at approximately 1 μm was prepared. We first investigated the effect of type-II quantum confinement associated with the GaSb/GaAs QRs from both the PL and time-resolved photoluminescence (TRPL) measurements of the unprocessed wafer. After then, a PhC circular nanobeam cavity was fabricated on the sample as a laser device. The cavity we demonstrated here possessed the advantages of both the PhC nanobeam cavity and microdisk cavity, which bring about a small modal volume and high Purcell factor nanocavity laser. The field distribution of lasing mode was computed with the three-dimensional finite-element method (3D-FEM), and the Purcell effect of the circular nanobeam cavity was investigated through the second-round TRPL after the fabrication. From the temperature dependency of lasing threshold, we found that the device exhibited a high characteristic temperature, signifying the high thermal stability introduced by type-II GaSb/GaAs QRs.

**Figure 1:**
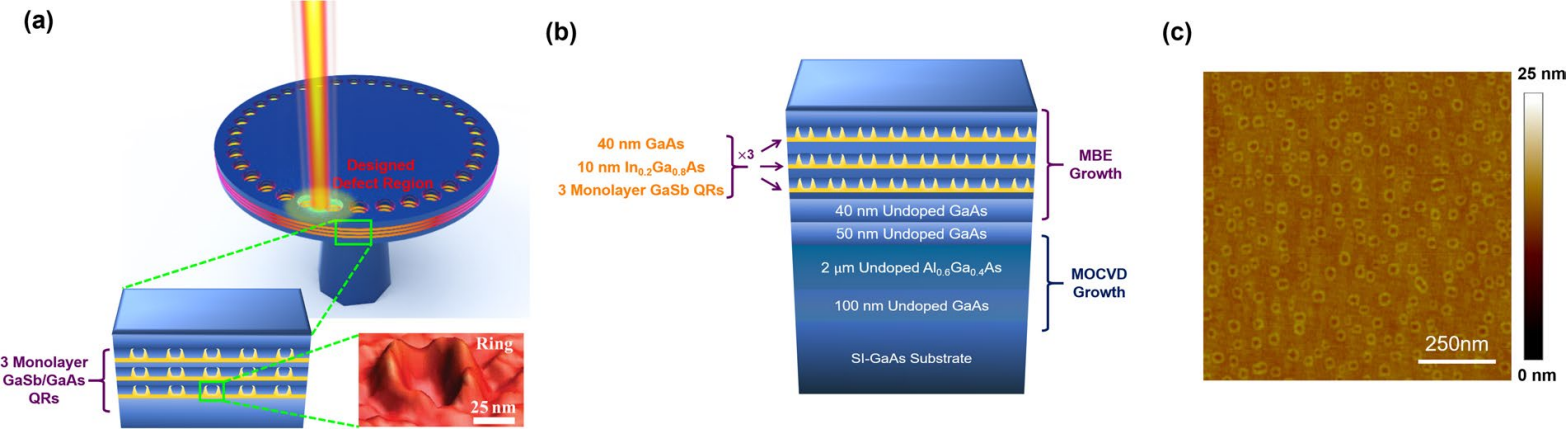
(**a**) Schematic of PhC circular nanobeam lasing with GaSb type-II QRs. (**b**) The layer structure of the GaSb/GaAs QR epitaxial wafer. (**c**) The AFM image of GaSb/GaAs QRs epitaxial wafer.

Figure 1(a) illustrates a schematic for the lasing of the PhC circular nanobeam cavity. The type-II GaSb/GaAs QRs acted as the gain medium, and the cavity was directly fabricated on the epitaxial wafer. The layer structure of the GaSb/GaAs QRs epitaxial wafer is shown in Fig. 1(b). The first half of the wafer includes a 2.0-μm-thick AlGaAs layer which was grown using the metal-organic chemical vapor deposition (MOCVD) on the GaAs substrate. For the second half, three GaSb QR single layers separated by InGaAs/GaAs barriers were formed by MBE^28,65^. The 3-single layers of QRs serve as the active region for photonic devices. Figure 1(c) shows the surface image of a GaSb/GaAs QRs single layer scanned with the atomic force microscope (AFM). The density of GaSb QRs was approximately 2.32 × 10^10^ cm^−2^, and the average height, inner and outer diameter of the rings were 1.5 nm, 23.0 nm and 46.7 nm, respectively.

**Figure 2:**
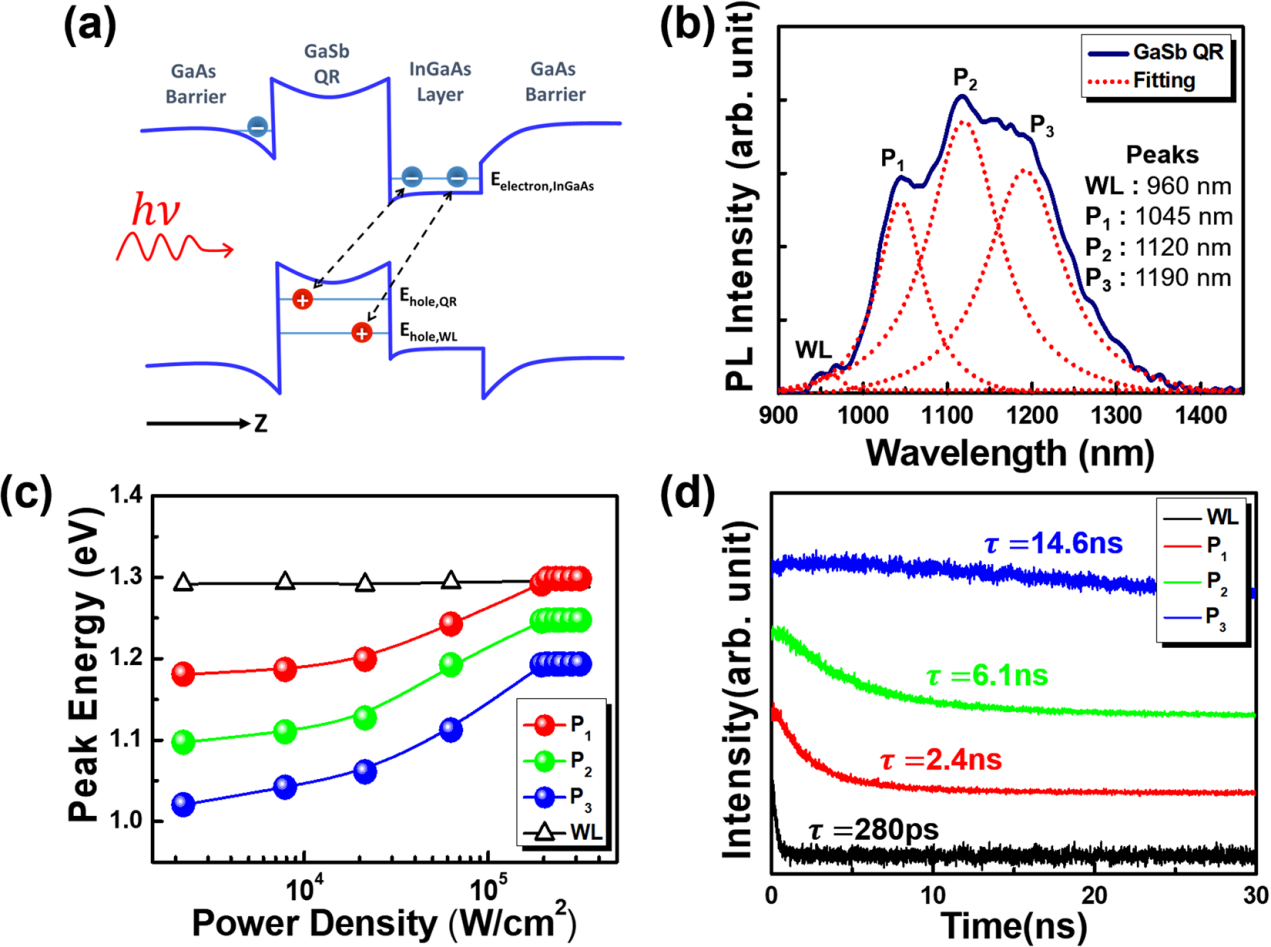
(**a**) The band diagram and carrier transition near a type-II GaSb/GaAs QR. (**b**) The PL spectrum from bulk GaSb/GaAs QRs at an excitation power density of 1.1 × 10^4^  W/cm^2^ and temperature of 80 K. (**c**) The blue shifts of PL peak energies as a function of the pumping power. (**d**) The time evolutions of TRPL from GaSb/GaAs QRs and WLs.

The optical characteristics of the unprocessed sample were first investigated. The schematic band diagram near a GaSb/GaAs QR in Fig. 2(a) illustrates the type-II quantum confinement and spatially indirect transitions of carriers. The spatial separation of electrons and holes around type-II nanostructures leads to weak wave function overlaps and lowers the probability of radiative recombination. Therefore, the radiative lifetime of type-II GaSb/GaAs QDs or QRs could be prolonged beyond the nanoseconds timescale^66^. On the other hand, the holes in bound valence states of QRs would still attract electrons toward the nanostructures with the Coulomb force. The interaction induced triangular potentials around the conduction band of GaAs and InGaAs regions, which weakly localized some of the electrons around the QRs and made the corresponding radiative transition rate higher than those of unbound electrons^30^. The weakly-bound electrons might also recombine with holes nonradiatively or simply escape from the nanostructure thermionically. Also, as the pumping intensity increased, the more populated holes in the QRs could steepen the triangular potential which localized the electrons. This squeezed the wave functions of the bound electrons and increased their energies. As a result, the energy levels of electron and holes shifted relative to each other, and the emission wavelength blue shifted could be observed^3,22,66,67^.

The PL from type-II GaSb/GaAs QRs was excited using an 850 nm continuous-wave diode laser and detected by an optical spectrum analyzer (OSA) below the room temperature (details see methods). Figure 2(b) shows the PL spectra from the QRs at 80 K at an excitation power density of 1.1 × 10^4^ W/cm^2^. Several broad spectral peaks corresponding to different ensembles of transitions were observed between 900 and 1400 nm. Applying the Gaussian fitting (dashed lines), we identified three prominent peaks (P_1_, P_2_, and P_3_) with a wavelength separation of approximately 70 nm (65–80 meV). A much weaker side lobe at the short-wavelength side was attributed to the emission from wetting layers (WLs). To further characterizing the emission properties of GaSb/GaAs QRs, pumping power dependent PL was investigated. As shown in Fig. 2(c), when the pumping power increased, the three spectral peaks P_1_, P_2_, and P_3_ blue-shifted significantly, reflecting an increasing number of electrons due to band filling and steepened triangular potential around QRs^3,68^. The blue shifts of peaks P_1_, P_2_, and P_3_ were proportional to the cube root of the pumping power, which is frequently been observed in type-II heterostructures and is also regarded as a characteristic of the steepened triangular potentials due to populated holes^4,6,28^. And the sub-linear pumping power dependence of P_1_, P_2_, and P_3_ peak intensity variation shown in Fig. S1 suggest the reduced carrier transfer in QRs (see supplementary information, section S1)^22^. These phenomenon support that the luminescence of peaks P_1_, P_2_, and P_3_ originates from the type-II band structure confinement in QRs^22,69,70^. On the other hand, the peak wavelength corresponding to the WLs remained almost the same as the excitation power increased, suggesting that this emission might not originate from the type-II quantum confinement. The experimental observation here was also consistent with the previous findings regarding GaSb/GaAs QDs^3,68^.

To clarify the emission dynamics of GaSb/GaAs QRs, TRPL measurements of the epitaxial wafer were performed at 80 K. The excitation source was an 860 nm pulsed diode laser operated at a repetition rate of 1 MHz and pulse width of 30 ps. The TRPL signals were collected with a NIR photomultiplier (PMT) detector and analyzed using a picosecond histogram-accumulating real-time processor (detail see method). The time evolutions of the TRPL signals at spectral peak wavelengths of WLs, P_1_, P_2_, and P_3_ are shown in Fig. 2(d). The decay traces were assumed to follow a single exponential decay function exp(−*t*/*τ*) of time *t*, where *τ* is the overall PL decay time that reflects the quench rate of carriers corresponding to different spectral peaks of type-II QRs^29^. The decay times τ of P_1_, P_2_, and P_3_ extracted from TRPL measurements were several nanoseconds, but the counterpart of WLs was only 280 ps. These numbers also support the speculation that the peaks P_1_, P_2_, and P_3_ originated from type-II QRs (relatively slow carrier recombination and leakage), whereas the short counterpart of WLs was caused by the fast escape in WLs.

The corresponding time evolution patterns of GaSb/GaAs QRs were similar to those reported for type-II GaSb QDs^6,27,66,71^. The shorter lifetime at the shorter peak wavelength could be a result of the faster escape of the carriers from the QRs due to the closer carrier energy between high energy ensembles and WLs peak. The three peaks may be connected to the discrete hole charging which are corresponding different number hole occupancies^22,27^. In PL spectra of the GaSb/GaAs QRs, the energy spacing was approximately two to three times larger than those previously reported^22,27^. The larger energy spacing might be due to the small height of QRs (around 1.5 nm) in the QRs. Moreover, the true energy spacing could be smaller because some ensembles might not be resolved due to thermal or other kinds of fluctuations.

**Figure 3:**
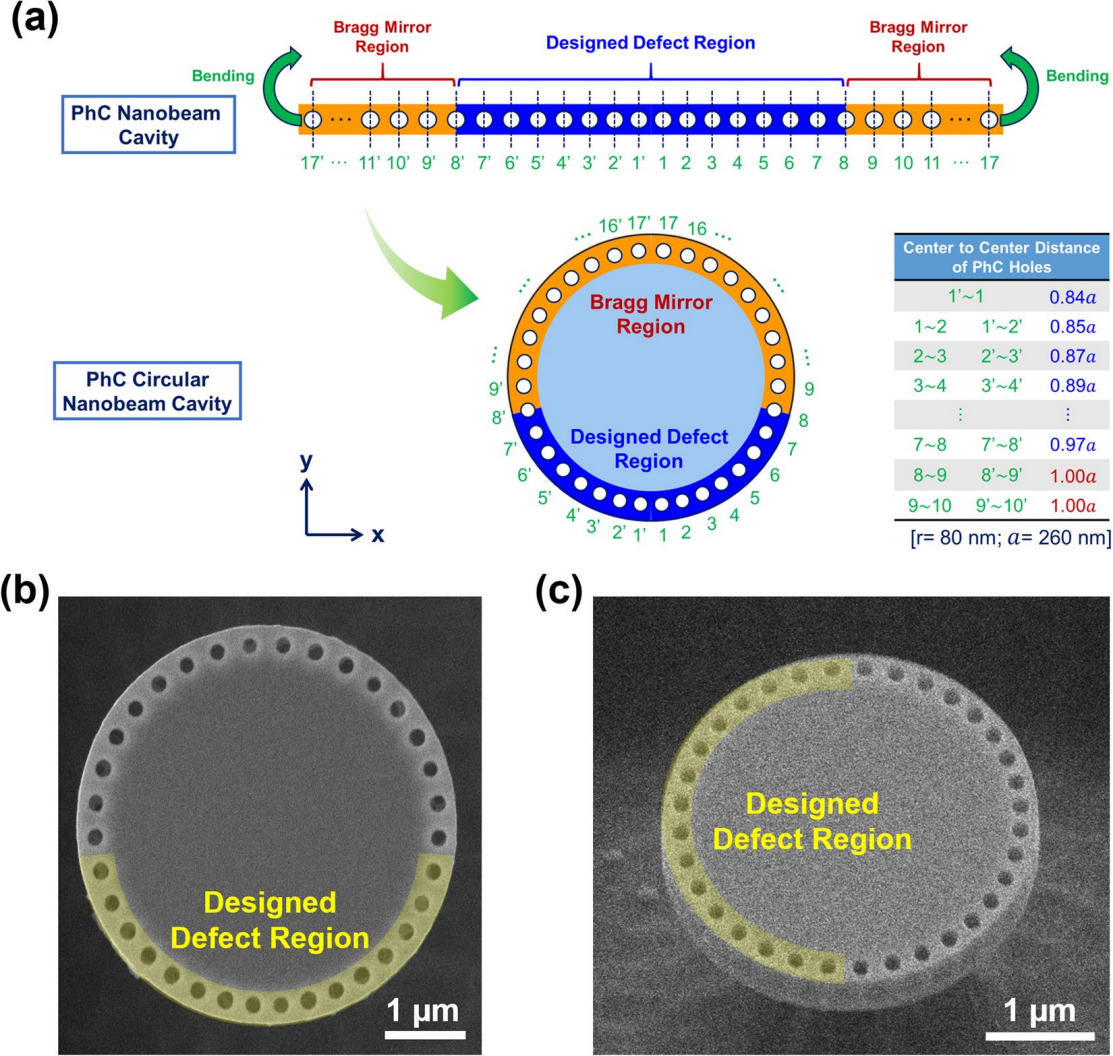
(**a**) Illustrations of a PhC nanobeam cavity virtually bent into a PhC circular nanobeam cavity. Various arc lengths of hole centers to the middle of the hole array in terms of a = 260 nm are listed in the table. The SEM images of the (**b**) top view and (**c**) tilt views of the fabricated PhC circular nanobeam cavity with an average lattice constant *a* around 260 nm and diameter of 3 μm.

In our previous work, we successfully demonstrated the lasing of type-II GaSb/GaAs QDs through the coupling to different types of micro and nanocavities^12,13^. The long carrier lifetime which is an intrinsic property of type-II quantum emitters can provide lasers with good power and thermal stability. In addition, it has been reported that the luminescence of GaSb/GaAs QRs could be stronger than that of QDs under specific circumstances^28–30^. It is promising and feasible to utilize GaSb/GaAs QRs as the gain medium of NIR nanolasers. As in our previous demonstration, we fabricated the laser cavity from a GaSb/GaAs QR epitaxial wafer in this study. As shown in Fig. 3(a), in the design of cavity, we conceptually bent a PhC nanobeam cavity into a circular one around the microdisk, that is, a PhC circular nanobeam cavity^41^. The periodic Bragg mirrors of the PhC nanobeam with a designed defect region were placed at the perimeter of the microdisk. These settings brought about two advantages. First, the intrinsic whispering gallery mode from the microdisk is turned into a localized defect mode of the nanobeam cavity (see supplementary information, section S2). Second, when a line-shape nanobeam cavity bend into the circular nanobeam cavity, the original Bragg mirrors region in each end of nanobeam cavity can be overlapped into one section, which could reduce the total length of the nanobeam cavity. The Bragg mirror can act as the mirror for both clockwise and counter-clockwise directions. Therefore, the optical confinement was further improved. Furthermore, this effect benefits reducing the total numbers of holes in actual device, thereby minimizing the device footprint.

For our design schematically shown in Fig. 3(a), the neighboring distances of the 16 holes were slightly tuned with their diameters unaltered. Adjusting the position of the holes potentially led to the formation of defect modes localized near certain parts of the hole array (defect regions). The center-to-center arc lengths of the hole in the hole array are listed in the unit of lattice constant *a* of the unbent nanobeam cavity. The lattice constant was fixed at *a* = 260 nm in our design, and the radius of holes were approximately 80 nm. The diameter and thickness of the microdisk were 3 μm and 240 nm, respectively.

The PhC circular nanobeam cavities were fabricated in a 240-nm-thick membrane that contained three GaSb/GaAs QR layers. Prior to the fabrication of cavities, we defined the PhC circular nanobeam pattern on the same epitaxial wafer analyzed above and followed by dry-etching (transferring pattern to active region) and wet-etching (creating the suspended structure) processes (details see methods). The top and tilt views of the scanning electron microscope (SEM) images of a fabricated PhC cavity are shown in Fig. 3(b,c), respectively.

**Figure 4:**
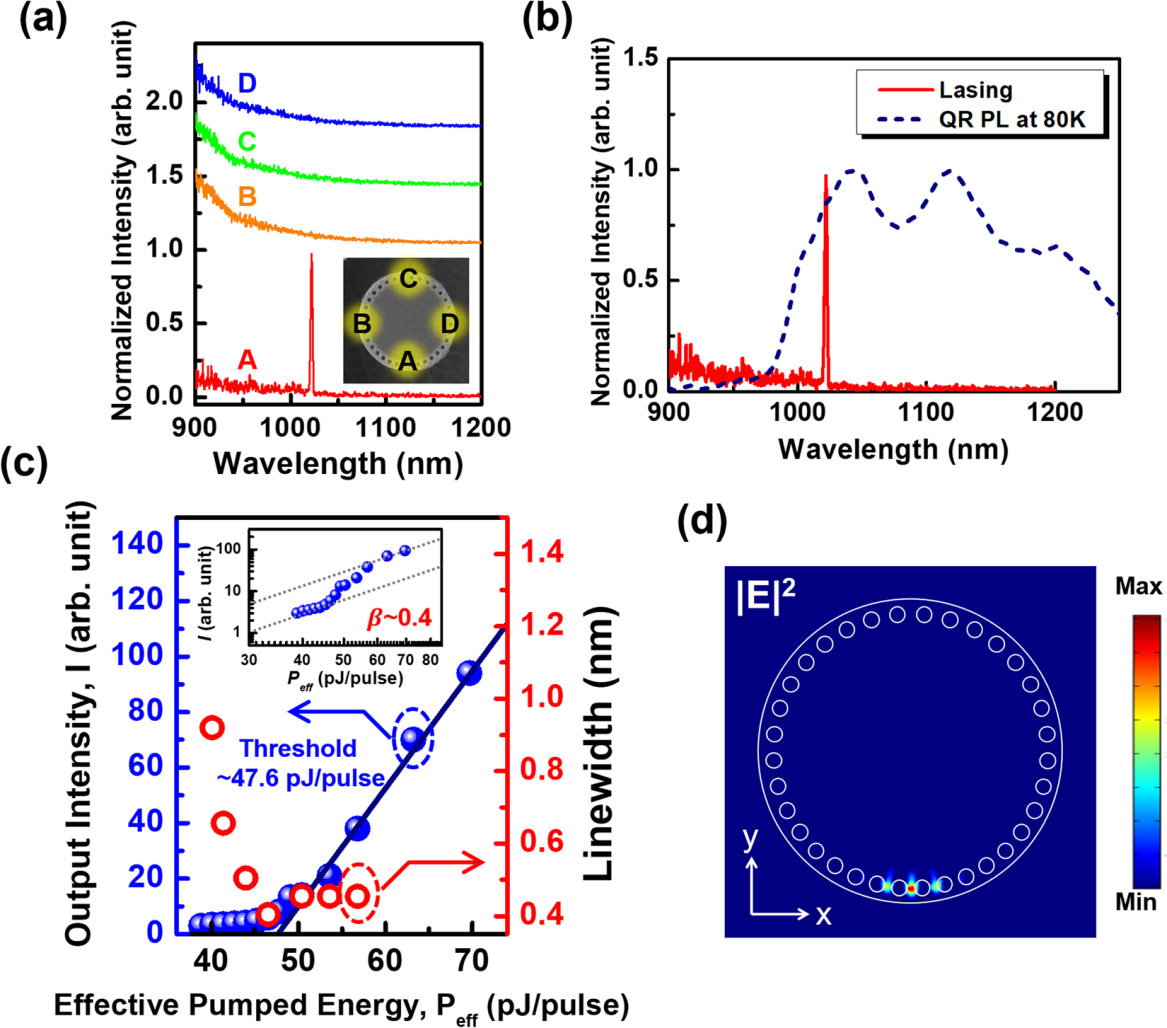
(**a**) The lasing spectra from the PhC circular nanobeam laser at 80 K. The lasing wavelength is 1022 nm. The inset shows pumping positions A, B, C, and D. Lasing is observed only at the designed defect region (Position A) at a lasing wavelength of 1022 nm. (**b**) The comparison between lasing spectrum with the PL spectrum of QRs at similar pumping power. (**c**) The light-in–light-out curve (blue dots) and linewidth variation (red dots) of the PhC circular nanobeam laser. Its effective threshold pumping energy was approximately 47.6 pJ/pulse. The insert figure shows the logarithmic-scale light-in-light-out curve. The experimental spontaneous emission coupling factor (β) value is approximately 0.4 for the PhC cavity laser. (**d**) The top view of the 3D-FEM calculated |E |^2^ profile of the defect mode in this PhC circular nanobeam cavity. The theoretical resonant wavelength is 1067.3 nm.

To investigate lasing characteristics of the PhC circular nanobeam cavity with type-II GaSb/GaAs QRs, the fabricated devices were optically pumped with an 850 nm diode laser in the cryostat at 80 K. The pumping diode laser was operated at pulsed mode with 0.5 MHz of reputation rate and 30 ns of pulse width. An OSA was used for the lasing signals collection from the PhC circular nanobeam cavity (details see method). The lasing action of the PhC circular nanobeam cavity was observed as the optical pumping power was increased. The lasing spectrum of the PhC circular nanobeam laser shown in Fig. 4(a) was obtained under effective pumping energy of around 60 pJ/pulse. The lasing peak appeared at a wavelength of 1022 nm with a linewidth of 0.45 nm, corresponding to quality factor (Q) of about 2200. Figure 4(c) illustrated the light-in–light-out (L–L) curve and linewidth variation under the different pumping power. The effective pumping energy was estimated by considering the overlapping between pumping laser spot, cavity geometry and the gain medium distribution. The laser had a low threshold pumping energy of approximately 47.6 pJ/pulse (corresponding to ~378.8 μJ/cm^2^ · pulse). It should be noted that this effective lasing threshold is in the same order as several reported PhC nanobeam cavity lasers based on type-I confinement quantum well^41,48^. We also compare the lasing spectrum with the PL of the unprocessed QRs sample which are shown in Fig. 4(b). It shows the resonant wavelength of the defect mode at 1022 nm lies in the spectral range of P_1_, which may correspond to the radiative transitions involving the excited hole states of the QRs. Populating the excited hole states, often requires a high pumping intensity, which rendered the lasing action inefficient. The lasing action from the P_2_ and P_3_ emissions of the QRs could be achieved with the different PhC cavity design. A new design which shifts the resonant wavelength of defect mode to the spectral ranges of P_2_ and P_3_ may lower the threshold and is expected to reduce the necessary excitation power for lasing.

To investigate the modal profile of the lasing mode in this cavity, the optical spectra corresponding to different pumping positions (A, B, C, and D indicated in the inset of Fig. 4(a)) were taken while keeping other pumping conditions unchanged. As shown in Fig. 4(a), the lasing action was only observed as the pumping beam was focused on Position A, which is the defect region of PhC circular nanobeam cavity. No signs of lasing were observed as the pumping spot was placed to other positions. To understand the origin of position-dependent phenomena, we carried out 3D-FEM calculations of the PhC circular nanobeam cavity based on the geometry of the fabricated cavity. A defect mode with its theoretical resonant wavelength at 1067.3 nm was obtained from FEM calculations. Figure 4(d) shows the electric field profile |E |^2^  of this mode (top view). The corresponding field distribution was nearly confined to the defect region (Position A). This defect mode was expected to be the lasing mode of the PhC circular nanobeam cavity. The pumping at position A whose excitation pattern overlapped well with the localized profile of the defect mode naturally selected it out. The slight deviation of the lasing wavelength (<5%) between the calculation and the experimental data was attributed to the fabrication imperfections. With 3D-FEM simulation, the modal volume of the lasing mode was calculated as 0.81 (λ/n)^3^ (see supplementary information, section S4) which is compatible with reported lasers with similar cavity design^41,72^. It is worth to note that there are more than one high-Q modes in the PhC nanobeam cavity. However, the resonant wavelength of the 2nd resonant mode is approximate 40 nm shorter than calculated 1st resonant mode which is out of the QRs gain region, we did not observe the lasing action from the 2nd resonant mode.

**Figure 5:**
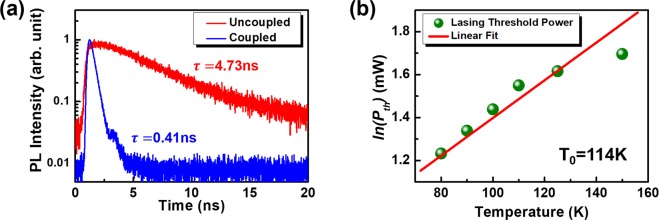
(**a**) TRPL spectra of the GaSb/GaAs QRs with (blue) and without (red) the coupling to the PhC circular nanobeam cavity at 80 K. The wavelength of the measurements was fix at 80 K 1022 nm (lasing wavelength). (**b**) The threshold power of the PhC circular nanobeam laser (logarithmic scale) at different temperatures.

The small modal volume of the lasing mode in the PhC circular nanobeam cavity lead a high Purcell factor (*F*)^73^ which would significantly modified the spontaneous emission in the cavity. Combined the simulated mode profile and the experimental quality factor, the theoretical Purcell factor 207 was obtained. With the fabricated devices, the experimental Purcell factor value can be estimated with the lifetimes of the carriers obtained through the TRPL measurement in the presence (or absence) of the PhC circular nanobeam cavity. The factor *F* was estimated as follows^55^:

1 $$F\approx \frac{{\tau }_{{\rm{QR}}}}{{\tau }_{{\rm{couple}}}}$$

where τ_QR_ and τ_couple_ are the decay times of the carriers around the QRs in an unprocessed sample and in the fabricated cavity (coupled with the cavity mode), respectively. The nonradiative process (quenching) was neglected because it should be considerably slower than the radiative process at low temperatures. In addition, for the luminescence off the cavity resonance, we saw not significant variation of the intensity, indicating that the surface recombination introduced by the fabrication did not play an essential role here. The time evolutions of TRPL of generic GaSb QRs and cavity-coupled counterparts at the lasing wavelength are shown in Fig. 5(a). The lifetime τ_QR_ was 4.73 ns, whereas the lifetime τ_couple_ was only 0.41 ns. Based on (1), the Purcell factor *F* was approximately 11.5, which marked the enhancement of the spontaneous emission rate in the nanobeam cavity^73^. This enhancement factor was quite significant for semiconductor lasers based on type-II QR gain materials.

There were several issued on the thermal stability and temperature-limited performance of micro or nanocavity lasers. The heat generated under laser excitation would enhance the nonradiative recombination of carriers, such as Auger recombination and surface recombination. These processes compete with the radiative recombination, reduce the stimulated emissions, and also increase the threshold power after the long-term operation of the device. It has been experimentally shown that the nonradiative Auger recombination could be suppressed in lasers with type-II quantum confined heterostructure^61–64^. We have reported a microdisk laser with type-II GaSb/GaAs QDs with a high threshold characteristic temperature of 77 K in our previous work^12^. With a similar approach to that in previous work, we have investigated the thermal characteristic of our PhC circular nanobeam laser which contained type-II GaSb/GaAs QRs as the gain material. The lasing pumping threshold power variation was analyzed at different environmental temperature. Typically, the temperature dependent lasing threshold power ${P}_{th}(T)$ would increase exponentially with operating temperature *T* which can be expressed as

2 $${P}_{th}(T)={P}_{th0}\exp (T/{T}_{0})$$

where *T*_0_ is the characteristic temperature of laser threshold. In Fig. 5(b), the variation of the lasing threshold power is shown in the logarithmic scale with temperature. By linear fitting the curve, we obtained a threshold characteristic temperature *T*_0_ of 114 K for our PhC circular nanobeam laser, which was higher than that reported in our previous study^12,13^. This number was also better than those of conventional devices based on type-I nanostructures^74^. The high threshold characteristic temperature suggested that the lasing performances of the presented PhC circular nanobeam laser with type-II GaSb/GaAs QRs are more stable under different environment temperature. It is also worth to note that CW lasing action of GaSb/GaAs QRs at room temperature can be observed with the larger-sized high-Q cavities. However, in this work, we focused only on the smaller photonic crystal cavities for the ultra-small mode volume and lower lasing threshold. In the future, the room temperature CW operated sub-micrometer PhC cavity laser with type-II QRs can be expected by placing laser cavities on the top of the high thermal conductive substrates^75–78^.

In summary, a PhC circular nanobeam laser based on type-II GaSb/GaAs QRs was demonstrated. Clear blue shifts of the spectral peaks on the PL spectra with increasing pumping power were observed for these QRs, revealing their type-II characteristics. The long-lived emission from GaSb/GaAs QRs and short-lifetime WLs were distinguished through TRPL measurements. Integrated the QRs with designed PhC circular nanobeam cavity which had a resonant wavelength of 1022 nm, we demonstrated the lasing action at a temperature of 80 K with quality factor around 2200. The lasing mode is a defect resonance of PhC circular nanobeam, which is strongly localized at the designed region. The nature of the mode was verified by the phenomenon of position-dependent pumping and 3D-FEM calculations. The PhC circular nanobeam cavity enhanced the spontaneous emission rate of the type-II GaSb/GaAs QRs by 11.5 times. Furthermore, the laser exhibits a characteristic temperature of 114 K. The lasing performances from the type-II GaSb/GaAs QRs are indeed relatively less efficient, compare to emission from the type-I III-V gain materials, due to the type-II band alignment. However, the emission from the type-II materials also exhibit the longer photon lifetime and might benefit to some unique applications and systems.

## Methods

### Devices fabrication

The presented PhC circular nanobeam cavities were directly fabricated on the epitaxial wafer containing GaSb/GaAs QRs as shown in Fig. 1(b). A 2.0-μm-thick AlGaAs layer was first grown with a MOCVD system followed by MBE growth 3-single layers of GaSb/GaAs QRs on GaAs substrate, the details of epitaxy parameters were similar to our previous reports^28,65^. Prior to the fabrication of the PhC circular nanobeam cavities, silicon nitride (Si_3_N_4_) and polymethyl methacrylate (PMMA) layers were deposited and spin-coated as an etched hard mask and a lithography mask, respectively, for successive processing steps. The PhC circular nanobeam patterns were defined using the electron beam lithography at 30 keV followed by two dry-etching steps using the mixture gases of CHF_3_/O_2_ (transferring pattern to the Si_3_N_4_ hard mask) and Ar/SiCl_4_ (transferring pattern to the active region of the epitaxial wafer) in the inductively coupled plasma (ICP) etching system. To form the suspended membrane, the Al_0.6_Ga_0.4_As sacrificed layer was partially removed with the HF solution (HF:H_2_O = 1:4) to form the post under the PhC circular nanobeam cavity.

### Optical characterization

The low temperature PL and TRPL spectra presented in this work were all performed in a home-built micro-PL system equipped with a cryostat. For the PL and lasing measurement, a transistor-transistor logic (TTL) modulated 850 nm diode laser was used as the excitation source which can be operated at continuous-wave mode for measuring the PL of QRs and switched to pulse mode with a repetition rate of 0.5 MHz and pulse width of 30 ns for characterizing the lasing performance of cavities. The excitation laser was focused by a 100× long-working-distance near-IR (NIR) objective (NA 0.5) with a beam spot ~4 μm in diameter. The PL and lasing signals were collected by the same objective lens and detected by an optical spectrum analyzer (OSA). For TRPL measurement, the excitation source was an 860 nm pulsed diode laser which operated at a repetition rate of 1 MHz and pulse width of 30 ps. The TRPL signals were collected using the same objective lens but detected with a NIR monochromator connected PMT detector module (Hamamatsu H10330A) and analyzed with a picosecond histogram-accumulating real-time processor (PicoQuant PicoHarp 300).

## Supplementary information

Supplementary information.
