# Supplementary material for: Photonic Crystal Circular Nanobeam Cavity Laser with Type-II GaSb/GaAs Quantum Rings as Gain Material
Source: Sci Rep. 2020 Mar 16;10:4757. doi: 10.1038/s41598-020-61539-5 (PMC7075894; doi:10.1038/s41598-020-61539-5)
Supplement: Supplementary file 1 — Supplementary information. [file 41598_2020_61539_MOESM1_ESM.docx]

***Supplementary Information***

**Photonic Crystal Circular Nanobeam Cavity Laser with Type-II GaSb/GaAs Quantum Rings as Gain Material**

*Hsiang-Ting Lin^1^, Kung-Shu Hsu^1,2^, Chih-Chi Chang^1,2^,* *Wei-Hsun Lin^1^,**Shih-Yen Lin^1, 2, 3^, Shu-Wei Chang^1,2^, Yia-Chung Chang^1,2^, and Min-Hsiung Shih**^1, 2, 4*^*

^1^Research Center for Applied Sciences (RCAS), Academia Sinica, Taipei 11529, Taiwan

^2^Department of Photonics and Institute of Electro-optical Engineering, National Chiao Tung University, Hsinchu 30010, Taiwan

^3^Graduate Institute of Electronics Engineering, National Taiwan University, Taipei 10617, Taiwan

^4^Department of Photonics, National Sun Yat-sen University, Kaohsiung 80424, Taiwan

**^*^Correspondence to e-mail address:** mhshih@gate.sinica.edu.tw

**S1. Possible Origins of the Sub-peaks in GaSb/GaAs PL Spectrum**

The excited state of electrons could be a possible cause to the three sub-peaks (P_1_, P_2_, and P_3_) observed in the PL spectrum. However, those electrons are only bound to QRs by the relatively weak attraction from holes inside the QRs, which may not support excited states with large energy spacing. Besides, Figure S1 shows the pump power dependent PL peaks intensity variations of GaSb/GaAs QRs. The sub-linear power dependences at higher pump power suggest the reduced carrier transfer in QRs^1^.

Alternatively, the spectral peaks may originate from the excited subbands of the InGaAs capping layer, which is effectively a quantum well (QW). However, the carrier relaxation in continuum states of a QW is fast, and at a temperature of 80 K, electrons tend to occupy the lowest subbands, which could not explain the presence of three peaks which were already present at the low pumping intensity. According to previous reports, these ensembles in GaSb/GaAs QRs PL are more likely due to the discrete hole charging, which each peak represents different number hole occupancies^1,2^.

In fact, one could make a rough estimation of the energy spacing 𝛥𝐸 of holes states of the GaSb QRs as follows:

$$\Delta E\approx\frac{\hbar^{2}}{2m_{V}^{*}}\left( \frac{\pi}{L} \right)^{2}$$

where $\hbar$ is the Planck constant; $m_{V}^{*}$ is the effective mass of holes; and 𝐿 is some characteristic length of the QR. Taking the heavy-hole effective mass $m_{V}^{*}$ =0.5$m_{0}$ for GaSb and a length of 𝐿 around 3 to 5 nm (the size of an island in QRs, see the inset of Figure 1a, we obtain an energy spacing 𝛥𝐸 ranging from 30 to 83 meV, which looks reasonable as compared to the experimental data (65-80 meV) shown in Figure 2b.

Figure S1. The GaSb/GaAs QRs PL peaks intensity variation with pumping power density which show sub-linear pumping power dependent.

**S2. Analysis of Resonant Modes in Photonic Crystal Circular Nanobeam Cavities**

The design of our photonic crystal (PhC) circular nanobeam cavities which combine a microdisk cavity with a PhC nanobeam cavity is similar as the demonstration by Zhang et al^3^. To investigate the resonances, we first analyze the whispering gallery modes (WGMs) in a 3 μm diameter and 240 nm thick microdisk. In this microdisk cavity, it supports degenerate TE_1,16_ WGMs at around 1300 nm which correspond to clockwise and counterclockwise propagated resonances along the circumference. Next, 32 holes with identical 260 nm hole-to hole angular spacing were arranged at the perimeter of the microdisk. These holes create periodic refractive index variation as a one-dimensional PhC nanobeam structure distributed along the circumference. It brings the degenerated TE_1,16_ WGMs split into two standing wave resonances whose H_z_ field concentrated in holes (dielectric band edge mode) and dielectric regions (air band edge mode) as shown in Figure S2(b) and Figure S2(c). Similar as the resonances in PhC structures, a bandgap between these band edges forbid WGMs propagated in. In addition, the corresponding band edge resonant wavelength vary as a function of the hole radius providing a sizeable bandgap as the 3D finite element simulation (3D-FEM) calculated results presented in Figure S2(a).

We choose the dielectric band edge mode with 80 nm hole radius to further design circular PhC nanobeam cavity because of two reason: 1) High overlapping between the electric field and gain medium of dielectric band edge modes are more suitable for lasing. 2) It shows the highest calculated quality factor of the design with 80 nm of hole radius as shown in Figure S2(d). Next, the designed defect region as descried in Figure 3(a) were introduced in the microdisk. The modified hole-to-hole angular spacing supports the defect resonance in the bandgap and the 3D-FEM calculated electric field profile shown in Figure 4(d) represent the significant mode localization in the designed defect region as conventional nanobeam cavities^4–7^.


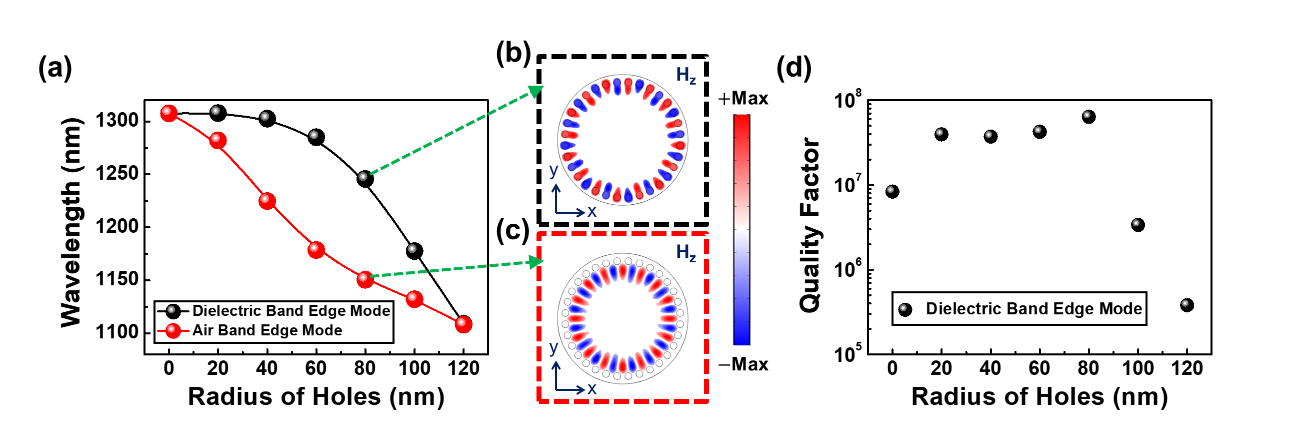


**Figure S2.** (a) Band edge resonant wavelength variation as a function of holes radius on circular PhC cavity without defect. The H_z_ field distribution of (b) dielectric band edge mode and (c) air band edge mode. (d) The calculated quality factors of dielectric band edge mode variation as a function of holes radius on circular PhC cavity without defect.

**S3. Optical Modes Confinement in QRs Layers**

The overlap between field and carriers in the active region (GaSb / GaAs QRs layers) is critical to lasing properties such as lasing threshold. This effect can be estimated by calculated the optical confinement factor (*Γ*) value of the laser cavity in numerical simulation following this definition:

$\Gamma=\frac{{\int\varepsilon_{QR}\left| \boldsymbol{E}_{\boldsymbol{QR}} \right|}^{2}dV}{\int_{-\infty}^{+\infty} \varepsilon\left| \boldsymbol{E} \right|^{2}dV}$

where $\varepsilon$ represents the dielectric constant, and *E* is the corresponding electric field in every position. The active region of 3 layers of QR (~11 nm thickness of each layer includes GaSb QRs and GaAs capping layer) in the middle of cavity were considered in simulation model. Applied above analysis, *Γ* of our laser is ~13.1%. The optical mode confinement in cavity is shown in Figure S3.


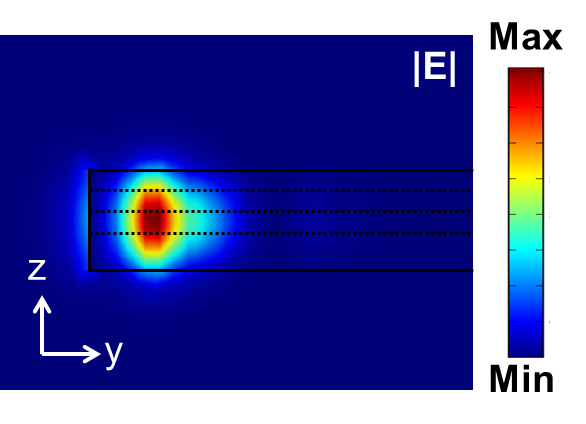


**Figure S3.** Side view of the electric field distribution at resonance for the circular PhC nanobeam cavity. The dashed line indicates the location of three QRs layers.

**S4. Estimation of Mode Volume and Purcell Factor**

One significant advantage of PhC nanobeam cavity lasers are their ultra-small mode volumes which lead high Purcell factors. The Purcell factor can be estimated by the following equation:

$$F=\frac{3Q}{4\pi^{2}V}$$

where Q is the quality factor of lasing resonance and V is the mode volume of resonance. Here, Q ~ 2200 is obtained from the measurement lasing spectrum, *V* can be calculated from numerical simulation by the definition $V=\frac{{\int\varepsilon_{Cavity}\left| \boldsymbol{E} \right|}^{2}dV_{Cavity}}{\max\left( \varepsilon\left| \boldsymbol{E} \right|^{2} \right)}\sim0.81\left( \frac{\lambda}{n} \right)^{3}$ where $\varepsilon$ is the dielectric constant, $\left| \boldsymbol{E} \right|$ is the electric field strength, $\lambda$= 1022 nm is the experimental resonant wavelength and n = 3.4 is the refractive index of GaAs. Therefore, the relatively small *V* lead a high theorical maxima $F$ ~ 207.

**S5. Estimation of Gain and Loss in Cavity**

The energy stored in an optical cavity which decaying exponentially with time $t$ and can be expressed as:

$$I\left( t \right)=I_{0}\exp\left( -\delta\cdot L \right)=I_{0}\exp\left( -\frac{t}{t_{c}} \right)$$

where $\delta$ is the loss factor during total round-trip length $L$ propagation, $t_{c}$is the photon lifetime in the cavity and can be described by quality factor *Q* and frequency $\omega$ followed by the definition as

$$t_{c}=\frac{Q}{\omega}=\frac{Q\lambda}{2\pi c}$$

When photon in the cavity propagating one round-trip with optical path length $L=n_{eff}\cdot L_{c}$ and time $t=t_{r}=\frac{n_{eff} L_{c}}{c}$, the energy in the cavity will reduce to

$$I\left( t_{r} \right)=I_{0}\exp\left( -\frac{2\pi n_{eff}}{Q\lambda}L_{c} \right)$$

Thus, the loss factor during one round-trip propagation can be defined as

$$\delta_{c}=\frac{2\pi n_{eff}}{Q\lambda}$$

For an active optical cavity, the lasing pumping threshold reached when the material gain $\Gamma g_{th}$ compensate cavity loss factor $\delta_{c}$,

$$\Gamma g_{th}=\delta_{c}=\frac{2\pi n_{eff}}{Q\lambda}$$

In our PhC circular nanobeam cavity coupled with GaSb/GaAs QRs, lasing occurred at $\lambda$ = 1022 nm with $Q$ ~2200 and $n_{eff}$ ~3.17. Therefore, the material gain $\Gamma g_{th}$ is estimated as 88.7 $\mathrm{cm}^{-1}$. It should be noted this value is compatible with the material gain of several semiconductor laser based on type-I InAs QDs as gain material^8,9^. And the corresponding modal gain $g_{th}$ is $6.72\times{10}^{2}$cm^-1^.

**References**

1. Hodgson, P. D. *et al.* Blueshifts of the emission energy in type-II quantum dot and quantum ring nanostructures. *Journal of Applied Physics* **114**, 073519 (2013).

2. Young, R. J. *et al.* Optical observation of single-carrier charging in type-II quantum ring ensembles. *Applied Physics Letters* **100**, 1–5 (2012).

3. Zhang, Y. *et al.* Photonic crystal disk lasers. *Optics Letters* **36**, 2704–2704 (2011).

4. Zhang, Y., McCutcheon, M. W., Burgess, I. B. & Loncar, M. Ultra-high-Q TE/TM dual-polarized photonic crystal nanocavities. *Opt. Lett.* **34**, 2694 (2009).

5. Deotare, P. B., McCutcheon, M. W., Frank, I. W., Khan, M. & Lončar, M. High quality factor photonic crystal nanobeam cavities. *Appl. Phys. Lett.* **94**, 121106 (2009).

6. Ahn, B.-H. *et al.* One-dimensional parabolic-beam photonic crystal laser. *Opt. Express* **18**, 5654 (2010).

7. Gong, Y. *et al.* Nanobeam photonic crystal cavity quantum dot laser. *Opt. Express* **18**, 8781 (2010).

8. Kirstaedter, N. *et al.* Gain and differential gain of single layer InAs/GaAs quantum dot injection lasers. *Appl. Phys. Lett.* **69**, 1226–1228 (1996).

9. Gilfert, C., Ivanov, V., Oehl, N., Yacob, M. & Reithmaier, J. P. High gain 1.55 μm diode lasers based on InAs quantum dot like active regions. *Appl. Phys. Lett.* **98**, 201102 (2011).
